# Unraveling the relationship between white matter lesions in MRI and migraine: a systematic review

**DOI:** 10.1055/s-0045-1812886

**Published:** 2025-12-22

**Authors:** Verena Subtil Viuniski, Matheus de Lima Ruffini, Adolfo Moraes de Souza, Raquel Prates dos Santos, Davi Henrique Galvao Fonseca Ribeiro, Leandro Lisboa Faoro, Fabiano Reis, Renata Gomes Londero, Juliana Avila Duarte

**Affiliations:** 1 Universidade Federal do Rio Grande do Sul, Faculdade de Medicina, Departamento de Medicina Interna, Porto Alegre RS, Brazil.; 2 Universidade Estadual de Campinas, Faculdade de Ciências Médicas, Departamento de Clínica Médica, Disciplina de Medicina Interna e Semiologia, Campinas SP, Brazil.

**Keywords:** Migraine Disorders, Magnetic Resonance Imaging, White Matter, Neuroimaging

## Abstract

**Background:**

White matter hyperintensities (WMH) are commonly detected on brain magnetic resonance imaging (MRI) scans of migraine patients, but their clinical relevance and underlying mechanisms remain uncertain.

**Objective:**

To systematically review the relationship between WMH and migraine, focusing on prevalence, progression, and associations with clinical and demographic characteristics.

**Methods:**

We conducted a systematic review of observational studies published between 1990 and May 2025, including adult patients with migraine (with or without aura) who underwent brain MRIs with at least 1.5T scanners. Data extraction was performed by two independent reviewers, with disagreements resolved by a third. Study quality was assessed using the Newcastle-Ottawa scale for observational studies.

**Results:**

A total of 25 studies were included, comprising approximately 3,600 participants, of whom 1,725 had migraine. Most participants were women and reported age means or medians typically between 30 and 60 years. Frequently, WMHs were observed in migraine patients, particularly in those with aura, longer disease duration, and higher headache frequency. No consistent association was found between WMH and comorbidities. Significant heterogeneity in imaging protocols, lesion quantification methods, and study design limited data comparability and precluded meta-analysis.

**Conclusion:**

Migraine patients often present with WMHs, but their clinical significance remains unclear. Future studies should employ standardized MRI protocols, volumetric lesion quantification, and consistent migraine phenotyping to clarify its pathophysiological role in migraine and potential implications for diagnosis and management.

## INTRODUCTION

As fairly common as migraine can be in the general population, so are the challenges it proposes to the clinical practitioner and referred neurologist. 1 The imaging diagnostic tools are ever improving, and it is tempting to address a disease through them, especially in areas where misdiagnosis can lead to permanent impairment.

Usually, when there is clinical evidence of migraine, as stated by the International Classification of Headache Disorders 3 ^rd^ edition (ICHD-3), 2 and no new features or neurologic impairment, there is no imperative need of a neuroimaging study to establish a diagnosis. 3

In outpatient settings, patients often seek medical help for headaches accompanied by anxiety, prompting the use of imaging resources to rule out other conditions, even when the clinical diagnosis is clear. While this approach is understandable from a patient care perspective, it can lead to unnecessary expenses. As the American Headache Society guidelines 4 note, neuroimaging is generally unnecessary in migraine cases with a normal neurological exam and no atypical features or red flags, as incidental findings like white matter lesions (WMLs) or atrophy typically do not alter the diagnosis or management.

Migraines with aura (MA) can be mistaken for stroke or neurodegenerative diseases. A study by Vijiaratnam et al. 5 found that MA patients were more likely to undergo computed tomography (CT) and magnetic resonance imaging (MRI), with the latter revealing more prevalent white matter hyperintensities (WMHs) compared to patients without aura.

Therefore, the routine use of imaging can increase costs, patient anxiety, and lead to further unnecessary tests. It is crucial to balance its need with potential harms. Certain headache characteristics may still justify neuroimaging, such as rapid onset, changes in pattern, or a lack of therapeutic response. 6 7

Commonly, WMH can be observed in migraine patients, but their clinical significance remains unclear. Current models are speculative, and further research is needed to establish causality. 7 8 9 There is an urge to establish such causal correlation, and other studies have attempted to address it in previous revisions and meta-analysis, 10 11 12 but none exclusively focused on the WMH and their anatomical correlations, or course and duration of the disease. This systematic review attempts to provide the required data in medical literature, to base such relation and prompt more investigation into this matter.

## METHODS

This review was conducted and reported in accordance with the Preferred Reporting Items for Systematic Reviews and Meta-Analyzes (PRISMA) 2020 guidelines. A protocol was registered on the PROSPERO platform (CRD42021265265). We included observational and cohort studies published between 1990 and May 2025 in indexed journals, investigating WMHs in patients with migraine, both with and without aura, aged over 16 years and without other neurological conditions, who underwent at least one brain MRI. The scan had to be performed on equipment with a minimum field strength of 1.5T.

Articles were retrieved from the Medical Literature Analysis and Retrieval System Online (MEDLINE/PubMed), Excerpta Medica (Embase), and Latin American and Caribbean Literature on Health Sciences (LILACS) databases using the search terms listed in **Supplementary Material 1** (available at https://www.arquivosdeneuropsiquiatria.org/wp-content/uploads/2025/09/ANP-2024.0351-Supplementary-Material-1.docx ), with the last search being conducted in May 2025.

**Table 1:** Selected studies general characteristics

Study	Design	Country of origin	MRI techniques	Covariates considered	N migraine	N MO / N MA	N control	N men migraine/control	Mean age (SD) migraine / control	Mean Follow-up (months)	WMH (basal / follow-up)
Trauninger et al. (2011) 16	Cross-sectional	Hungary	3T Siemens Trio Tim; WMH rated on T2WI and FLAIR	Disease duration, attack frequency, age, sex, smoking, thyroid function, cholesterol, uric acid	186	141/45	No control group	30	Migraine: 36.4 (8.9)	0	MO: 42 MA: 16 No follow-up, but number of patients with WMH increased with the increase of disease duration
Dinia et al. (2011) 17	Prospective longitudinal study	Italy	1.5T GE Signa Excite, FLAIR, FRFSE T2	smoking, hypertension, diabetes, hypercholesterolemia, coagulation disorders, right-to-left shunt, age	41	0/41	No control group	Migraine: 8	Migraine: mean age 41.8 ± 12.7 years; mean disease duration 16.9 ± 13.6 years	32.1 (mean follow-up for one set of MRI-detected WMLs), 33.4 (mean follow-up for another set of MRI-detected WMLs)	MA: 26/8 *no control group
Seneviratne et al. (2012) 31	Cross-sectional	Australia	1.5T GE MRI, FLAIR and DWI (no contrast)	Age, sex, headache frequency/month, migraine duration, family history, MA/MO, vascular risk factors	44	26/18	No control group	Migraine: 7	Migraine with WMH: 40.6 Migraine without WMH: 50.1	0	MO: 7 MA: 12 *no control, no follow-up, increased headache frequency was found to be the factor associated with WMH
Palm-Meinders et al. (2012) 18	Prospective longitudinal study	Netherlands	Not specified	Age, sex, center (Maastricht), education, BMI, hypertension, antihypertensive use, blood pressure, diabetes (self-reported), history of stroke/TIA, smoking (ever/current), pack-years, alcohol use, triptans, ergotamines, prophylactic use, oral contraceptives	203	89/114	83	Migraine: 58 Control: 26	Migraine: 57 (7.8) Control: 55 (7.3)	108	MO: 89/68 MA: 114/84
Hamedani et al. (2013) 19	Prospective longitudinal study	USA	1.5T MRI with axial 5 mm slices, WMH severity graded	Age, race/center, sex, diabetes, cholesterol, blood pressure, antihypertensive use, smoking, coronary disease history, BMI, alcohol, Income	93	60/33	935	ARIC total cohort: 44%	ARIC total cohort: mean 60 years	48	MO: 2.72 mL (20.40, 5.82) / 1.13 mL (21.20, 3.46) MA: 2.58 mL (21.67, 6.83) / 2.50 mL (20.70, 5.70) *lesional volume
Erdélyi-Bótor et al. (2014) 14	Prospective longitudinal study	Hungary	3T Siemens TIM Trio: T1WI, T2WI, FLAIR, DWI, PWI, 1H-MRS, T1/T2 relaxation	Age, sex, clinical migraine aspects, migraine type, disease duration	17	10/7	0	Migraine: 2	Migraine: 47.0 ± 11.2	36	MO: 10/9 MA: 7/7 Lesional volume: 0.896 mL (0.628–2.434) / 1.140 (0.842–2.666)
Honningsvåg et al. (2018) 28	Cross-sectional	Norway	1.5T GE Signa HDx, FLAIR, T1WI	Age, sex, employment, alcohol, daily smoking, self-rated health, ischemic heart disease, cerebral infarction, HADS, BMI, SBP, DBP, cholesterol, triglycerides, clucose	91	29/62	551 (no headache)	Migraine: 24 Control: 291	Migraine: 57.7 (4.3) Control: 58.8 (4.2)	0	407 patients with WMH (9 with migraine)
Lee et al. (2019) 15	Cross-sectional	South Korea	3T Philips Achieva: 3D T1WI and FLAIR, high-resolution protocol	MCA-BHI, age, sex, MA, BMI, headache days/month, vasoactive med use, depression, anxiety	86	71/15	35	Migraine: 21 Control: 10	Migraine: 37.3 (7.87) Control: 33.1 (8.72)	0	WMH number Migraine: 6.2 (9.80) Control: 4.6 (5.70) WMH volume, cm3 Migraine: 0.080 (0.1551) Control: 0.026 (0.0374)
Arkink et al. (2019) 24	Prospective longitudinal study	Netherlands	1.5T Philips Gyroscan Intera: MTI, dual-echo T2, FLAIR	Age, sex, education, BMI, SBP, DBP, hypertension, diabetes, smoking (ever/current), pack-years, cholesterol, HDL, prophylactic use, triptans, ergotamines, disease duration, migraine attacks/year, high attack frequency	137	58/79	74	Migraine: 40 Control: 25	Migraine: 49.0 (8.0) Control: 48.4 (8.0)	108	MO: 20/20 MA: 30/29
Kocatürk et al. (2020) 47	Cross-sectional	Turkey	1.5T Siemens MRI scanner	Age, sex, BMI, smoking, family history, migraine duration, attack frequency, attack duration, pain intensity (VAS), lipid profile, hemoglobin	75	75/0	30	Migraine: 15 Control: 9	Age (median): No WMH = 30.5 years With WMH = 36 years Controls = 31 years	0	Most migraine patients with WMH had 1–3 or >9 lesions, mainly in subcortical and frontal regions. No association was found between lesion burden and migraine severity (attack frequency, VAS, duration).
Alkhaffaf et al. (2020) 48	Prospective longitudinal study	Iraq	T1, T2, FLAIR, DWI with reported TR/TE	MIGSEV, pain intensity, attack duration, aura, nausea, disability, tolerability, frequency of attacks, treatment resistance	100	N/A	No control	Migraine: 44	WMHI-positive: 41.2 ± 8.2 years WMHI-negative: 31.6 ± 9.3 years	3	WMH at baseline: Identified in 36% of patients (36/100) — hyperintense WML, primarily diagnosed on T2/FLAIR images. Follow-up WMH: Not performed.
Dobrynina et al. (2021) 20	Cross-sectional	Russia	3T Siemens MRI scanner	Age, sex, migraine type, attack frequency, disease duration, pain intensity, vascular risk factors (hypertension, diabetes, obesity, hypercholesterolemia, smoking), oral contraceptive use, acute/prophylactic treatments, lab markers	92	56/36	24	Migraine: 19 Control: 4	34.6 ± 8.9 / 32.8 ± 6.9	0	WMH were observed in 41.0% of MO and 44.4% MA ( *p* = 0.991), and in 44.2% of EM and 38.7% of CM cases ( *p* = 0.379). Lesions were focal, median size 2.5 mm, located only in frontal, parietal, and temporal lobes.
Al-Hashel et al. (2022) 21	Cross-sectional	Kuwait	3T Siemens MRI scanner	Age, sex, migraine type, frequency, time since diagnosis, attack duration, homocysteine levels, symptomatic and prophylactic drug use, WMH location/laterality, MIDAS, HIT-6	60	48/12	20	Migraine: 10 Control: 3	38.65 ± 8.45 / 35.80 ± 8.20	0	WMH prevalence was 40% in migraine patients and 10% in controls. No association was found with attack duration or pain severity. WMHs were more frequent in CM and MA.
Ahmed et al. (2022) 32	Cross-sectional	Egypt	3T Philips MRI scanner	Age, sex, migraine type, frequency, time since diagnosis, attack duration, associated symptoms, drug use (symptomatic and prophylactic), WMH location/laterality, pain intensity (VAS)	500	350/150	0	Migraine: 270	Age (median): 36 years	0	WMHs were present in 29% of migraine patients, more common in older age, MA, and vomiting. They were associated with more severe migraine features and poorer response to both acute and preventive treatments, including larger lesions and higher Scheltens scores.
Imai et al. (2022) 33	Retrospective case-control study	Japan	Axial T1WI, FLAIR, DWI, MRA	Age, sex, migraine history (MA/MO), emotional stress trigger, thunderclap features, WMH characteristics, MRA findings, other brain lesions, drug treatments	50	39/11	37	Migraine: 5 Control: 10	Migraine: 40 ± 13 years Control: 38 ± 14 years	3	Basal WMH: DSWMH present in 46% of RCVS patients with migraine; 22% without Follow-up WMH: Progression of DSWMH in chronic stage found in 50% of RCVS patients with migraine; 22% without
Silva et al. (2023) 29	Cross-sectional	Brazil	3T MRI with VBM	Age, disease duration, aura frequency, attack frequency, pain intensity, prophylactic treatment, hypertension, diabetes, dyslipidemia, stroke, smoking, alcohol, obesity	45	15/15	15	Migraine: 0 Control: 0	MO = 33.00 ± 0.78 years MA = 33.86 ± 7.70 years CM = 32.53 ± 9.36 years Controls = 32.87 ± 9.68 years	0	Nearly 50% of participants had no WMLs. All lesions were supratentorial, predominantly in frontal and parietal lobes. No infratentorial lesions were observed. Mean WML volumes were MO: 26.0 mm ^3^ , MA: 13.7 mm ^3^ , CM: 16.8 mm ^3^ , controls: 19.2 mm ^3^ . Mean total WML counts were MO: 4.1, MA: 4.8, CM: 1.5, controls: 5.0. No significant group differences were found.
Antony et al. (2023) 27	Cross-sectional study	India	FLAIR, T2-weighted TSE, T1 sagittal SE, T1 post-contrast (axial, coronal, sagittal)	Age, sex, MIGSEV, pain intensity, attack duration, aura, nausea, disability, tolerability, frequency of attacks, treatment resistance	100	48/52	No control	Migraine: 41	Migraine patients: 28.60 ± 7.0 years No control group	0	WMH assessed: only at baseline using MRI. Follow-up WMH assessments: None conducted.
Iyigundogdu et al. (2023) 22	Cross-sectional study	Turkey	FLAIR axial, T1 axial, T2 axial/coronal/sagittal	Age, sex, smoking status, MA/MO, disease duration, attack frequency/duration, hematological markers (MPV, PDW, NLR, platelets, leukocytes, neutrophils, lymphocytes), WMH features	218	184/34	No control	Migraine: 34	Patients with WMH: median 42.5 years (IQR: 36–47) Patients without WMH: median 32 years (IQR: 26–41.25)	0	Basal WMH: 48 patients (22.0%). Follow-up WMH: Not performed; only baseline assessment was conducted.
Meilán et al. (2023) 23	Cross-sectional study	Spain	FLAIR, dual-echo PD/T2, sagittal T1	Age, migraine type (chronic/episodic), vascular risk factors (hypertension, hypercholesterolemia, smoking, age >45), analgesic overuse, aura history, medication use (preventive/symptomatic), comorbidities (depression, fibromyalgia, hypothyroidism)	125	62/63	EM: 29 women (used as control for CM)	Migraine: 0 Control: 0	CM: mean 43 years (16–65) EM: mean 36 years (16–58)	0	Basal WMH: WMH were detected in 76 patients (60.8%): 59 with CM (61.5%) and 17 with EM (58.6%). Most lesions were small, deep, and located in the frontal lobes. dWMLs: observed in 63% of CM and 71% of EM patients. pWMLs: found in 22.9% of CM and 17.2% of EM patients. Follow-up: Not performed.
Schramm et al. (2024) 25	Retrospective longitudinal study	Germany	3T Siemens MRI with FLAIR for WMH visualization	Age, sex, smoking (current/past), BMI, education, blood pressure, diabetes, cholesterol (LDL, HDL)	Visit 1: 398 Visit 2: 152	Visit 1: 286 / 112 Visit 2: 109 / 43	Visit 1: 169 Visit 2: 23	Visit 1 total (Migraine + Control): 582 Visit 2 total (Migraine + Control): 218	Visit 1 Migraine men: 55.9 ± 12.8 Migraine women: 59.1 ± 11.7 Control men: 65.6 ± 11.7 Control women: 66.5 ± 8.2	44	WMH volume was higher with age, with medians of 4,005 mm ^3^ in women and 4,812 mm ^3^ in men. Women with headaches had 1.23× more WMH than those without. WMH progression was greater in women (+238 mm ^3^ ) than in men (+109 mm ^3^ ). Migraine was not associated with higher WMH volume, progression, or Fazekas scores.
Nanda et al. (2024) 34	Cross-sectional study	India	3T MRI with DTI, 64 directions, b = 1200 s/mm ^2^	Age, sex, migraine type, disease duration, medication use, headache frequency	30	MO: 86.6%, MA: 13.3%	20 controls	Migraine: 3 Control: 0	Majority of patients in the age group of 20–40 years	0	Found in 36.7% of patients at initial evaluation; no mention of follow-up data specifically for WMH
Ali et al. (2025) 26	Cross-sectional	Netherlands	3T Philips MRI, semiautomated WMH segmentation confirmed by rater	Age, BMI, smoking, menopause, oral contraceptive use	38	0 / 38	35	Migraine: 0 Control: 0	Migraine: 51.3 (4.9) Control: 52.2 (5.0)	0	Basal only: MA (group 2): 0.81 mL (SD: 1.11 mL) Group with neither stroke nor migraine (group 4): 0.68 mL (SD: 0.92 mL)
Liu et al. (2025) 35	Cross-sectional	China	3T MRI, conventional sequences: T2-FLAIR, T2WI	Age, sex, WMH location, image quality, lesion boundary clarity, number of lesions, artifact presence	50	Not explicitly detailed	N/A	Migraine: 22	30 years (SD not specified)	0	The study investigates white matter hyperintensities (WMHs) at baseline; follow-up data are not provided
Abdalla et al. (2025) 36	Cross-sectional	Jordan	3T MRI, sequences: T1, T2, FLAIR	Age, sex, incidental findings	670	Not explicitly detailed	N/A	Migraine: 160	Migraine: 40.3 ± 14.5 years	0	Detected in 46.1% of patients; no follow-up imaging reported
Sun et al. (2025) 30	Cross-sectional observational study	China	7T Siemens MRI: sMRI (3D T1), fMRI, DTI	Age, sex, BMI, education, headache days/month, migraine days/month, attack duration, VAS, laterality, exercise aggravation, nausea, vomiting, photophobia, phonophobia, analgesic use, analgesic type, PHQ-9, GAD-7, PSQI	44	44 / 0	19 CM – MOH; 25 CM + MOH; 19 healthy controls.	Migraine: 0 Control: 0	Migraine without medication overuse: 39.11 ± 11.25 Migraine with medication overuse: 41.76 ± 12. 33 Control: 39.37 ± 11.82	0	DTI revealed reduced FA in the left cingulum bundle of CM + MOH patients, indicating greater white matter microstructural damage compared to CM − MOH. This suggests that medication overuse may worsen WM integrity.

Abbreviations: 1H-MRS, proton magnetic resonance spectroscopy; ARIC, atherosclerosis risk in communities; CM, chronic migraines; DBP, diastolic blood pressure; DSWMH, deep and subcortical white matter hyperintensity; DTI, diffusion tensor imaging; DWI, diffusion-weighted imaging; dWMLs, deep white matter lesions; EM, episodic migraines; FA, fractional anisotropy; FLAIR, fluid attenuated inversion recovery; FRFSE, fast-recovery fast spin-echo; GE, gradient-echo; HADS, Hospital Anxiety and Depression Scale; HDL, high density lipoprotein; HIT-6, Headache Impact Test-6; IQR, interquartile range; LDL, low density lipoprotein; MA, migraine with aura; MCA-BHI, Breath-Holding Index of the bilateral middle cerebral arteries; MIDAS, Migraine Disability Assessment; MIGSEV, migraine severity; MO, migraine without aura; MOH, medication overuse headache; MPV, mean platelet volume; MRI, magnetic resonance imaging; MTI, magnetization transfer imaging; NLR, neutrophil-to-lymphocyte; PDW, platelet distribution width; pWMLs, periventricular white matter lesions; RCVS, reversible cerebral vasoconstriction syndrome; SBP, systolic blood pressure; SD, standard deviation; T1WI, T1 weighted image; T2WI, T2 weighted image; TIA, transient ischemic attack; TSE, turbo spin echo; VAS, visual analogue scale; VBM, voxel-based morphometry; WMH, white matter hyperintensities; WMHL, white matter hyperintense lesions; WML, white matter lesions.

Two independent reviewers screened titles and abstracts using the Rayyan (Qatar Foundation) platform, with disagreements resolved by a third reviewer. Data extraction was performed and compared by two investigators, and disagreements were solved by a third reviewer. Missing or unclear data were documented and, when necessary, the corresponding authors were contacted for clarification.

The extracted data included the number of subjects, gender, mean age, number of patients with and without aura, the number of subjects with WMH in MRI, and lesion volume in milliliters. Additional data included disease duration, headache frequency, and follow-up information. Studies were evaluated using the Newcastle-Ottawa Scale (NOS) for observational studies ( **Supplementary Material 2** , https://www.arquivosdeneuropsiquiatria.org/wp-content/uploads/2025/09/ANP-2024.0351-Supplementary-Material-2.docx ), including an adapted version for cross-sectional designs. 13 Due to the methodological and statistical variability across the studies, a meta-analysis was not feasible. As a result, the findings were synthesized qualitatively.

**Table 2:** Selected studies' strengths and limitations

Study	Strengths	Limitations
Trauninger et al. (2011) 16	Assessment and statistical analysis of risk factors associated with WMH; Evaluation for WMH in both MA and MO separately.	Small sample size; Few details on MRI acquisition protocol and WMH measurement; No control group, no follow-up.
Dinia et al. (2011) 17	Well established MA diagnosis and documented disease course/duration; Assessment and report of comorbidities; Longitudinal design allowing follow-up MRI of all the participants.	Small sample size; Nongenetic laboratory parameters were only assessed at the baseline; No volumetric analysis of the WMH; Did not consider MO patients; No control group.
Seneviratne et al. (2012) 31	Reported the distribution of WMH load in the brain; Exploited potential for confusion with MS and clues for the distinction with migraine lesions.	Small sample size; No control group Retrospective design Possible selection bias (severe and complicated migraine).
Palm-Meinders et al. (2012) 18	Longitudinal design; Length of follow-up; Well characterized cohort; ICHD criteria for migraine diagnosis; Sensitive and reproductible methods of MRI acquisition.	Wide CI; Possible selection bias: ⅓ of the original baseline population was not reinvestigated.
Hamedani et al. (2013) 19	Large, biracial, populational-based cohort; Standardized headache and WMH assessment.	Possible selection bias: the brain MRI subcohort was smaller; Headache assessment was retrospective and did not use ICHD criteria; Possible dilution effect: patients were treated for migraine and may be on the migraine-free group; Unknown duration of disease and crisis per year.
Erdélyi-Bótor et al. (2014) 14	Through description of the MRI acquisition protocol; Volumetric measurement of all lesions Reported the distribution of the WMH lesions on the brain.	Small sample size; Lack of follow-up images of control group.
Honningsvåg et al. (2017) 28	Large population-based design; Assessment of headache status at two times; Reproducible MRI protocol and manual volumetry.	Possible selection bias: participants with lower CV risk; No gender stratification; Self assessment of headache (did not use ICHD).
Lee et al. (2019) 15	Use of a high-sensitivity automated quantification method to detect WMH (DEWS); Clinical and neurophysiological assessment of all participants.	Different protocol for MRI acquisition between patients and controls; No sex and age matching.
Arkink et al. (2019) 24	Well documented and reproductible MRI acquisition protocol; Long follow-up; Age, sex, and comorbidities adjusted.	Small sample size; Possible selection bias: loss of over half of the participants on follow-up.
Kocatürk et al. (2020) 47	Specific group, standardized WMH measures, midlife focus, control/stroke comparison.	Cross-sectional, no follow-up, limited migraine details, small sample, no cognitive tests.
Alkhaffaf et al. (2020) 48	Advanced MRI (Syn T2-FLAIR), lesion visibility by region, quantitative comparison.	Small sample, no aura data, cross-sectional, possible MRI bias.
Dobrynina et al. (2021) 20	Large sample, MRI by two readers, assessed age/gender impact on WMH.	Cross-sectional, limited clinical correlation, possible confounding.
Al-Hashel et al. (2022) 21	High-res 7T MRI, multimodal imaging, focused on females, analyzed medication overuse.	Cross-sectional, female-only, no follow-up, small sample.
Ahmed et al. (2022) 32	Large population-based sample, longitudinal, stratified by sex, robust MRI.	Definitions varied, limited young adults, possible group heterogeneity.
Imai et al. (2022) 33	Used DTI, analyzed microstructural changes in episodic/chronic migraine.	Small sample, cross-sectional, no dynamic WMH data.
Silva et al. (2023) 29	MA/MO groups, controlled comorbidities, manual MRI analysis.	Small sample, manual review, no follow-up.
Antony et al. (2023) 27	Compared migraine types, excluded vascular risks, stratified analysis.	Small sample, cross-sectional, no follow-up.
Iyigundogdu et al. (2023) 22	3T MRI, group division by subtype, brain region-specific analysis.	Small sample, no follow-up, no control group.
Meilán et al. (2023) 23	Linked WMH to carotid thickness, highlighted need for CV monitoring.	Small sample, aura-only, no follow-up.
Schramm et al. (2024) 25	Good sample, acute vs. prophylactic treatment, MA vs. MO.	No control, no comorbidity exclusion, no follow-up.
Nanda et al. (2024) 34	Novel regional study, n = 100, severity scales, gadolinium MRI, blinded reading.	Single-center, no control, short duration, selection bias.
Ali et al. (2025) 26	Long-term follow-up, defined criteria, regression analysis, serial imaging.	Retrospective, no blinding, limited generalizability, no recurrence data.
Liu et al. (2025) 35	Large sample, hematologic and neuroimaging data, ICHD diagnosis.	Cross-sectional, no control, no timing control for labs, no thrombophilia markers.
Abdalla et al. (2025) 36	Blinded imaging, detailed WMH characterization, EM comparison, adjusted analysis.	No healthy control, small EM group, frequent treatments, no follow-up.
Sun et al. (2025) 30	Prospective, standardized scales, uniform treatment, excluded confounders.	Small sample, no aura data, no WMH longitudinal tracking, subjective measures, no control group.

Abbreviations: CI, confidence interval; CV, cardiovascular; DEWS, deep white matter hyperintensity segmentation; DTI, diffusion tensor imaging; ICHD, International Classification of Headache Disorders; MA, migraine with aura; MO, migraine without aura; MRI, magnetic resonance imaging; WMH, white matter hyperintensities.

## RESULTS

**Figure 1:**
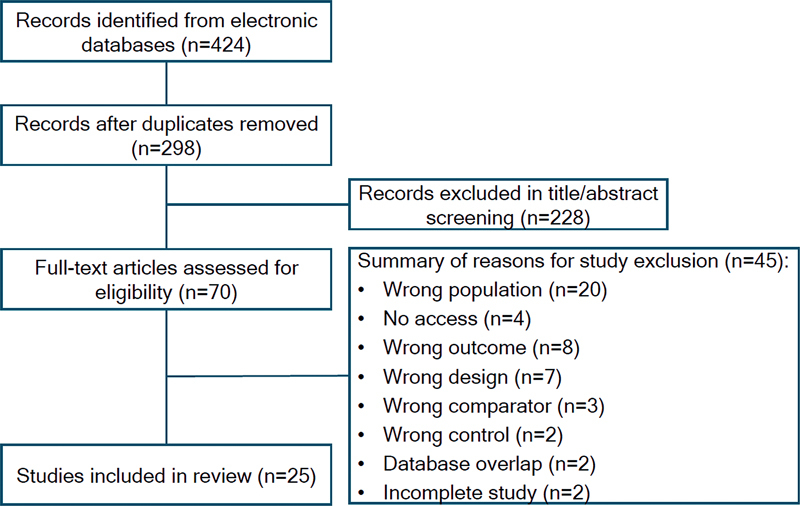
Prisma flowchart.

The search on the aforementioned platforms initially retrieved 424 studies. After manual exclusion of duplicates, 298 unique records remained. Two independent reviewers screened the titles and abstracts, excluding 228 records based on predefined criteria. Any discrepancies were resolved by a third reviewer. A total of 70 full-text articles were then assessed for eligibility, resulting in the exclusion of 45 with justification. Ultimately, 25 studies met the inclusion criteria and were part of the final analysis, with their characteristics presented in Table 1 . The study selection process is detailed in Figure 1 .

The strengths and weaknesses of each study can be found in Table 2 and will be further discussed. There was a considerable amount of missing data, and we tried to reach the authors of two studies but did not receive replies in 6 months. The study populations' heterogeneity, the different machines and imaging methods, and mostly the difference in measuring outcomes (such as lesion volume) led to the realization that performing meta-analysis with this data was not the best practice.

Finally, most studies were performed with a small sample of participants, and for that reason their isolated results are not applicable for all populations. Only two studies had a large population-based design, 14 15 but their data were also limited due to possible selection bias.

### Participant characteristics

The included studies encompassed a total of approximately 3,600 participants, including 1,725 individuals with migraine and 1,878 control participants. The populations were predominantly composed of adults, with migraine subtypes including migraine without aura (MO), migraine with aura (MA), and chronic migraine (CM). Sample sizes varied widely, ranging from small, well-characterized clinical cohorts to large population-based samples.

Most studies reported a higher proportion of female participants, consistent with the epidemiology of migraine. Ages ranged from young to older adults, with reported means or medians typically between 30 and 60 years. Several studies provided age distributions stratified by presence of WMH, migraine subtype, or control status.

Approximately half of the studies included a control group, which varied in definition, from headache-free individuals to general population controls. Most studies applied standardized diagnostic criteria, such as those from the International Classification of Headache Disorders (ICHD). Some studies reported clinical features, including disease duration, attack frequency, comorbidities (e.g., cardiovascular risk factors), and aura status.

Several studies included in this review explored the relationship between comorbidities—particularly cardiovascular and metabolic risk factors—and WMHs in migraine patients. Trauninger et al. 16 reported a possible association between it and elevated cholesterol, uric acid, and markers of endothelial dysfunction. Dinia et al. 17 found WMH in 26 of 41 patients (63%) but did not identify significant associations with vascular risk factors, likely due to limited statistical power. Palm-Meinders et al., 18 in a Dutch cohort of 203 migraine patients and 83 controls, assessed multiple comorbidities, including hypertension, diabetes, and lipid levels, but found no consistent association with WMH burden. Hamedani et al., 19 using data from 93 migraine patients and 935 controls within the Atherosclerosis Risk in Communities (ARIC) study, emphasized the challenges of identifying robust associations given the multifactorial nature of WMH and the complexity of adjusting for confounders.

Other studies, including those by Dobrynina et al. 20 (n = 92), Al-Hashel et al. 21 (n = 60), Iyigundogdu et al. 22 (n = 218), and Meilán et al. 23 (n = 125), also collected data on cardiovascular risk factors, such as hypertension, diabetes, hyperlipidemia, obesity, and smoking. However, their findings regarding comorbidity associations were either not statistically conclusive or not the primary focus of analysis.

Overall, while comorbidities were frequently considered in study designs, the evidence remains heterogeneous and inconclusive. Larger, standardized, and statistically powered studies are needed to clarify the role of cardiovascular and systemic risk factors in the development of WMH among migraine patients.

### Imaging protocols and analysis of WMHs

Most studies utilized accessible and widely available MRI protocols, primarily incorporating T2-weighted and FLAIR sequences, which allow for reliable WMH visualization. However, important limitations remain due to heterogeneity in image acquisition parameters, lesion definition, and quantification approaches. While many studies reported the presence or severity of WMH, only a subset performed volumetric measurements.

Studies such as Palm-Meinders et al., 18 Arkink et al., 24 Schramm et al., 25 and Lee et al. 15 quantified WMH volume with consistent methodology and reported distribution across brain regions. Among these, only Palm-Meinders et al. and Arkink et al. included both control groups and follow-up volumetry, enhancing the validity of their longitudinal comparisons.

Several studies employed advanced segmentation strategies. Palm-Meinders et al., 18 Arkink et al., 24 and Lee et al. 15 used automated or semi-automated methods for WMH detection, often combined with manual correction. Lee et al. notably introduced the deep white matter hyperintensity segmentation (DEWS) framework, a machine learning-based algorithm validated for high-sensitivity WMH detection in younger populations. Similarly, Ali et al. 26 applied semi-automated segmentation with expert validation, enhancing reproducibility. Schramm et al. 25 provided both volumetric and Fazekas scoring, offering robust multi-dimensional assessments of WMH burden.

On the other hand, several studies relied on manual visual assessment without volumetric quantification, including Dinia et al., 17 Erdélyi-Bótor et al., 14 Antony et al., 27 and Iyigundogdu et al., 22 which may introduce interobserver variability and limit comparability across studies. Visual scales, such as Fazekas and Scheltens, were applied in studies like Honningsvåg et al., 28 while Silva et al. 29 employed voxel-based morphometry (VBM), and Sun et al. 30 utilized a high-resolution 7T multimodal MRI, combining structural, diffusion tensor imaging (DTI), and fMRI data to characterize WMH microstructure.

Despite methodological advancements, challenges persist. Many studies lacked control groups, and few stratified findings by migraine subtype or reported interrater reliability. The diversity of approaches—ranging from visual inspection to artificial intelligence-based segmentation—reflects both innovation and a need for standardization. Future research would benefit from harmonized imaging protocols, consistent volumetric quantification, and systematic reporting of WMH distribution, to facilitate pooled analyses and improve comparability across studies.

### Findings on migraine patients' white matter

The main findings across the included studies were heterogeneous, although most shared the goal of evaluating the burden, distribution, and progression of WMHs in individuals with migraine. Most studies reported WMHs as supratentorial, small, and located predominantly in deep frontal white matter, with prevalence rates varying considerably across cohorts.

Trauninger et al. 16 found no significant difference in WMH frequency between migraine with and without aura and observed that lesions were more frequent in patients with longer disease duration and higher attack frequency. Dinia et al. 17 observed WMHs in 63.4% of migraine with aura patients at baseline, with 19.5% developing new lesions after a 33-month follow-up. Seneviratne et al. 31 reported WMHs in 43.2% of patients and identified age, family history of migraine, and attack frequency as associated factors. In the community-based study by Palm-Meinders et al., 18 women with migraine, especially those without aura, had higher WMH volume and more lesion progression than controls, independently of comorbidities. Similarly, Hamedani et al., 19 using longitudinal data from the ARIC cohort, found that migraine without aura was associated with greater WMH burden, although this association was not significant after full adjustment for confounders.

Longitudinal imaging by Erdélyi-Bótor et al. 14 showed that WMHs increased in number over three years, though lesion progression was not associated with clinical variables such as age or disease duration. The Nord-Trøndelag Health (HUNT) MRI study by Honningsvåg et al. 28 did not find a significant association between migraine and WMHs but did identify one with tension-type headache.

Lee et al. 15 found WMHs in nearly 70% of young migraine patients, although lesional burden was mild and only age correlated with volume. Arkink et al., 24 in a 9-year follow-up study, confirmed greater WMH volume progression in migraine patients compared with controls, particularly in those with aura.

Among more recent studies, Dobrynina et al. 20 reported WMHs in 42.4% of migraine patients, and Al-Hashel et al. 21 and Ahmed et al. 32 found similar rates of 40 and 29%, respectively. Imai et al., 33 studying migraine in patients with reversible cerebral vasoconstriction syndrome (RCVS), identified WMHs in 46% of cases. Silva et al. 29 reported WMHs in approximately 50% of migraine patients using VBM, while Antony et al. 27 and Iyigundogdu et al. 22 observed rates of 36 and 22%, respectively.

Meilán et al. 23 found WMHs in 60.8% of patients, distributed similarly between episodic and chronic migraine, Schramm et al. 25 observed a progressive increase in WMH volume in a large longitudinal cohort. However, migraine was not associated with higher volume or progression, as assessed by both volumetric analysis and Fazekas scoring.

Additional findings include Nanda et al. 34 reporting WMHs in 36.7% of patients, Ali et al. 26 identifying higher WMH volume in migraine with aura compared to controls, and Liu et al. 35 and Abdalla et al. 36 documenting WMHs in 22 and 46.1% of migraine patients, respectively. Finally, Sun et al. 30 used 7T multimodal MRI to reveal microstructural white matter alterations even in patients without visible WMHs on conventional sequences, highlighting advanced imaging's potential to detect subtle abnormalities.

In summary, despite differences in design, imaging methodology, and patient characteristics, most studies confirm that WMHs are frequently observed in migraine patients. However, the extent, location, and clinical implications of these lesions remain variable, reinforcing the need for standardized imaging protocols and longitudinal studies with rigorous clinical phenotyping.

## DISCUSSION

The objective of this systematic review was to analyze the relationship between WMH and migraine, focusing on their prevalence, progression, and potential associations with clinical and demographic factors. The key findings indicate that WMHs are frequently observed in migraine patients—particularly in those with MA, longer disease duration, and higher headache frequency. Prevalence rates among migraine patients varied across studies, ranging from 22 to over 60%, with some reporting small, deep, frontal WMHs as the most common pattern. 20 29 23 26

Previous studies have often overlooked essential migraine characteristics and the influence of comorbidities. While many explored WMH prevalence, few addressed their progression or clear associations with clinical subtypes, such as aura or attack frequency. Our review bridges this gap by synthesizing studies assessing both baseline and longitudinal data, with an emphasis on disease chronicity and migraine phenotypes. Notably, studies like Dinia et al. 17 and Erdélyi-Bótor et al. 14 provided longitudinal follow-up and identified WMH progression in a subset of patients.

Our findings align with Zhang et al., 37 who performed a meta-analysis of 30 studies and found a pooled WMH prevalence of 44% among migraineurs, with a higher prevalence in patients with aura. While their study highlighted associations with age and comorbidities like hypertension and diabetes, our synthesis did not confirm consistent correlations with these conditions, suggesting potential differences in methodology, population characteristics, or WMH detection techniques. Indeed, several studies in our review, such as Trauninger et al., 16 Palm-Meinders et al., 18 and Erdélyi-Bótor et al., 14 found no statistically significant influence of comorbidities on WMH presence or progression.

Masson et al. 38 added complementary evidence by examining structural brain changes beyond WMH, identifying alterations in both gray and white matter volume in migraine patients. Although our review specifically targeted WMH, their results support the hypothesis that migraine-related brain changes are multifaceted and may reflect broader anatomical or neurovascular dysfunctions. 38

Designing robust WMH studies requires careful control of confounding factors. Participants' mean age must be considered, as older individuals may naturally develop WMHs due to aging or vascular comorbidities. Using the latest ICHD criteria is essential for standardized migraine classification, and MRI acquisition protocols must be consistent across studies to ensure comparability. 39 40

One persistent challenge is the absence of a validated quantitative or qualitative scale tailored for WMH assessment in migraine patients. This gap, along with its historical perception as a benign condition, may contribute to the scarcity of large, representative studies. However, given the impact of migraines on quality of life and productivity, advancing our understanding of WMH is critical. With growing insights into migraine pathophysiology, particularly involving calcitonin gene-related peptide (CGRP) signaling, 41 42 a vascular component to WMH development appears plausible, although a direct mechanistic link remains speculative.

While Schramm et al. 25 did not find a strong association between migraine and WMH progression, our analysis highlights the WMH burden in older patients and those with longer migraine history. For example, Meilán et al. 23 observed WMHs in 60.8% of migraine patients, and Schramm et al. 33 reported volumetric progression over time. Other studies using advanced imaging techniques, like Sun et al. 30 with 7T MRI, demonstrated microstructural changes even in patients lacking visible WMH on conventional scans.

Interestingly, across multiple studies in our review, 16 17 18 14 comorbidities such as hypertension or diabetes were not associated with WMH presence or progression. This contrasts with broader epidemiological findings where such comorbidities are established risk factors, 43 44 45 indicating that WMH in migraine patients may follow distinct pathophysiological mechanisms.

This review underscores the need for future studies to investigate the cognitive and cerebrovascular implications of WMH in migraine. Although our focus was on lesion prevalence and progression, the long-term consequences of this condition remain poorly understood.

The strengths of our review include rigorous inclusion criteria and a comprehensive approach to both cross-sectional and longitudinal data. Limitations include heterogeneity in imaging methods, small sample sizes in some studies, and limited follow-up duration. Advancing knowledge on WMH in migraine will require coordinated efforts using standardized diagnostic criteria, quantitative imaging, and careful control of confounding variables.

In conclusion, across 25 studies (∼ 3,600 participants), WMHs are frequently observed in migraine, particularly in patients with aura, longer disease duration, and higher attack frequency. The strength of evidence is moderate and stems predominantly from cross-sectional designs, which consistently report higher WMH burden in migraine patients than in controls.

Evidence from prospective cohorts is inconsistent: some longitudinal studies show modest lesion accrual in subgroups, whereas others find no excess progression, so causality and prognostic significance remain uncertain. Findings from retrospective clinical samples generally echo the cross-sectional signal but are limited by referral bias, heterogeneous imaging protocols, and variable WMH definitions.

When present together, WMHs in migraine should be interpreted with caution: they are common incidental findings, typically small, supratentorial, and deep-frontal/parietal, and do not by themselves warrant changes in routine diagnostic workup or management in otherwise typical migraine presentations. Clinically, careful assessment is important to avoid overinvestigation and to assist in the differential diagnosis with demyelinating disease such as multiple sclerosis. 46

To clarify pathophysiology and clinical relevance, well-designed prospective studies are needed. This includes use of standardized MRI acquisition, consistent volumetric and topographic WMH quantification, and rigorous migraine phenotyping (ICHD), with adjustment for vascular/metabolic comorbidities and evaluation of cognitive and cerebrovascular outcomes. These improvements will enhance comparability across studies and support evidence-based counselling for migraine patients who present with WMHs.
